# Applying circuit theory and landscape linkage maps to reintroduction planning for California Condors

**DOI:** 10.1371/journal.pone.0226491

**Published:** 2019-12-31

**Authors:** Jesse D’Elia, Joseph Brandt, L. Joseph Burnett, Susan M. Haig, Jeff Hollenbeck, Steve Kirkland, Bruce G. Marcot, Arianna Punzalan, Christopher J. West, Tiana Williams-Claussen, Rachel Wolstenholme, Rich Young

**Affiliations:** 1 Pacific Regional Office, U.S. Fish and Wildlife Service, Portland, Oregon, United States of America; 2 California Condor Recovery Office, U.S. Fish and Wildlife Service, Ventura, California, United States of America; 3 Ventana Wildlife Society, Monterey, California, United States of America; 4 Forest and Rangeland Ecosystem Science Center, U.S. Geological Survey, Corvallis, Oregon, United States of America; 5 The Northwest Habitat Institute, Corvallis, Oregon, United States of America; 6 Pacific Northwest Research Station, U.S. Forest Service, Portland, Oregon, United States of America; 7 Department of Ecosystem Science and Sustainability, Colorado State University, Fort Collins, Colorado, United States of America; 8 Wildlife Program, Yurok Tribe, Klamath, California, United States of America; 9 Department of Wildlife, Humboldt State University, Arcata, California, United States of America; 10 Pinnacles National Park, U.S. National Park Service, Paicines, California, United States of America; University of Waikato, NEW ZEALAND

## Abstract

Conservation practitioners are increasingly looking to species translocations as a tool to recover imperiled taxa. Quantitative predictions of where animals are likely to move when released into new areas would allow managers to better address the social, institutional, and ecological dimensions of conservation translocations. Using >5 million California condor (*Gymnogyps californianus*) occurrence locations from 75 individuals, we developed and tested circuit-based models to predict condor movement away from release sites. We found that circuit-based models of electrical current were well calibrated to the distribution of condor movement data in southern and central California (continuous Boyce Index = 0.86 and 0.98, respectively). Model calibration was improved in southern California when additional nodes were added to the circuit to account for nesting and feeding areas, where condor movement densities were higher (continuous Boyce Index = 0.95). Circuit-based projections of electrical current around a proposed release site in northern California comported with the condor’s historical distribution and revealed that, initially, condor movements would likely be most concentrated in northwestern California and southwest Oregon. Landscape linkage maps, which incorporate information on landscape resistance, complement circuit-based models and aid in the identification of specific avenues for population connectivity or areas where movement between populations may be constrained. We found landscape linkages in the Coast Range and the Sierra Nevada provided the most connectivity to a proposed reintroduction site in northern California. Our methods are applicable to conservation translocations for other species and are flexible, allowing researchers to develop multiple competing hypotheses when there are uncertainties about landscape or social attractants, or uncertainties in the landscape conductance surface.

## Introduction

Endangered species reintroductions and translocations are important and increasingly applied conservation interventions to save species from extinction and facilitate their recovery [1, 2]; but, failure rates can be high when not properly planned [2–4]. For most species, a key determinant of success is identification of the reintroduction landscape, which considers ecological, social, and institutional dimensions [5, 6]. This can be particularly important for species that have large home ranges and those that have the potential to cause human-wildlife conflict.

*A priori* identification of the reintroduction landscape via modeling species’ movement probabilities away from release sites has important conservation implications for (1) defining areas where regulatory protections may be needed, (2) quantifying whether there will be sufficient ecological resources for the reintroduced population, (3) identifying and quantifying threats, (4) identifying barriers or restrictions to movement and colonization, and (5) predicting gene flow and future metapopulation connectivity. This is particularly critical for wide-ranging species where simply identifying breeding areas or local conditions in the immediate vicinity of the reintroduction site is insufficient. When paired with habitat linkage or population dynamics models, predicting movement probabilities away from reintroduction sites can also provide information necessary for identifying habitats in need of protection [6–8], comparing likely costs and outcomes of various reintroduction scenarios [9], and for helping to define and quantify recovery targets [10].

The California condor (*Gymnogyps californianus*) is a critically endangered species with a global population of <350 free-flying individuals. Ongoing efforts to restore the species to its former range in southern and central California, Baja California, and Arizona have been well-chronicled [11–13]. However, condors are still reliant on human intervention for survival and population growth primarily due to the continued use of lead ammunition, which condors ingest when scavenging on dead animals or gut piles left in the field [13, 14]. California condors remain absent from large portions of their historical range although planning efforts are underway to restore them to the Pacific Northwest via a new release site in northern California [15].

California condors are a good case-study for modeling the geographic extent of the reintroduction landscape because they can undertake long daily flights from nests or roosts to find carrion, and their movements have been closely monitored at multiple sites for many years [12, 16]. Around the proposed reintroduction site in northern California, nesting, roosting, and feeding habitats are relatively expansive and in close juxtaposition [17]. However, existing habitat models do not account for distances from release sites, habitat connectivity, nor landscape resistance to movement [17]. Furthermore, existing models that were trained and tested with data from condors using terrestrial features did not account for differences in activity-specific habitat selection by condors during flight [17]. Connectivity modeling using circuit theory [18] is one tool that has been used for assessing the likelihood of species’ recolonizing an area [19], identifying habitat connectivity between occupied sites and reintroduced populations [20], and for understanding why reintroduced populations have not colonized apparently suitable habitats [21]. We propose that circuit theory can also be useful in predicting the dispersion of condors from reintroduction sites.

Our goals were to: (1) develop a habitat model to predict landscape conductance (the inverse of landscape resistance) across the study area, based on the relative likelihood of occurrence of condors in flight around existing release sites in southern and central California; (2) use electrical circuit theory and our model of landscape conductance to empirically derive and test a spatially-explicit working hypothesis delineating the area of predicted landscape occupancy around release sites, and (3) map habitat connectivity between modeled core nesting areas around the proposed northern California release site and existing release sites. We expect our model of condor movement probabilities around a new reintroduction site to be useful for recovery planning and intend to test and update this model as we acquire new movement data. Our methods are broadly applicable to conservation translocations for other species where sufficient data exists to produce a reliable landscape conductance surface.

## Methods

### Study area

**Fig 1:**
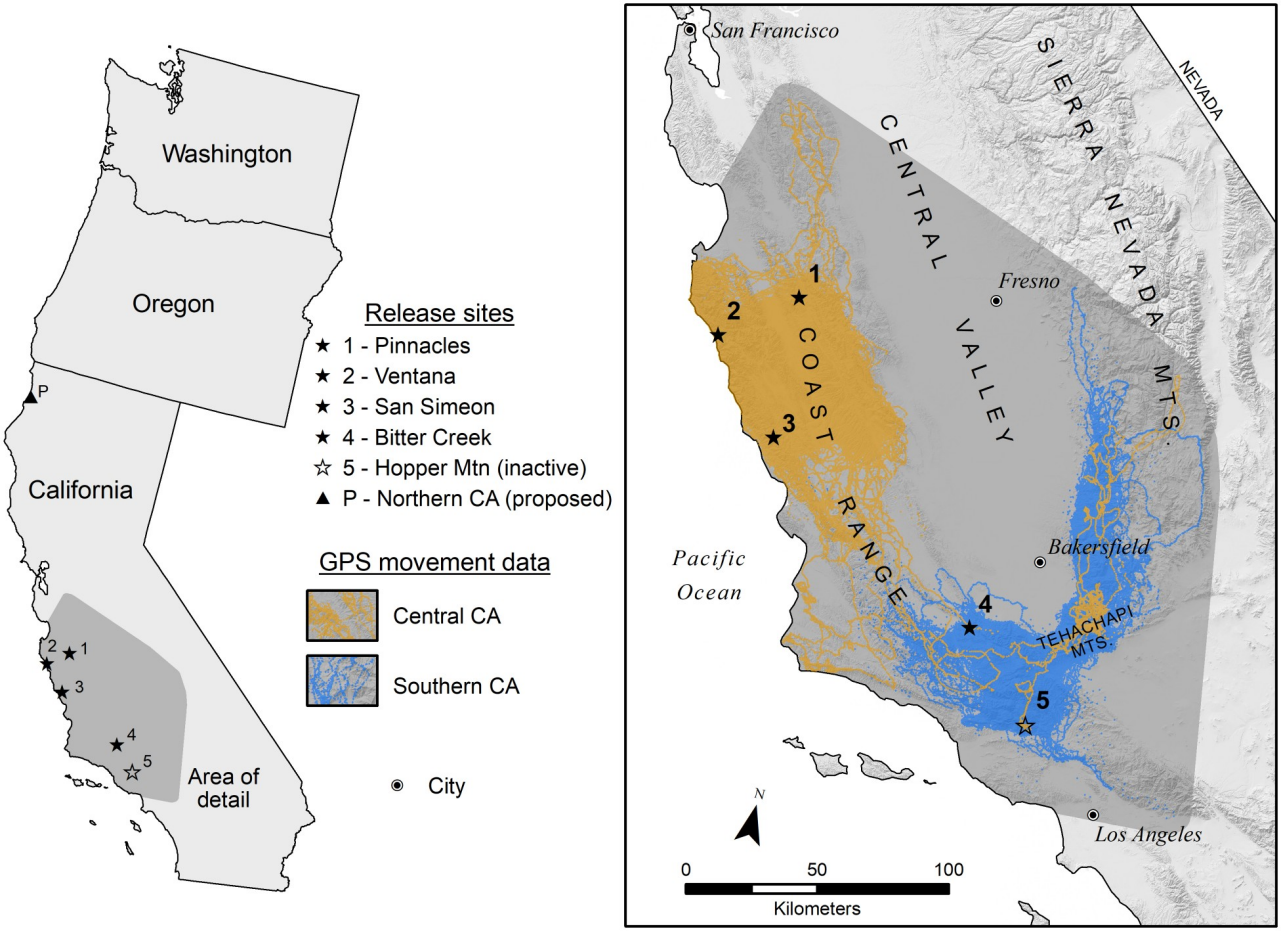
Study area, background area for developing a California condor landscape conductance surface (dark gray), in-flight California condor locations (July 2013-May 2017), and release sites.

Our study area included California, Oregon, and Washington, USA (32°-49°N and 114°-124°W). Within the study area, the current range of the California condor is limited to the mountainous regions of southern and central California where reintroductions are ongoing at four release sites: one in southern California operated by the U.S. Fish and Wildlife Service, two in central California along the Pacific coast operated by Ventana Wildlife Society, and one in central California operated by Pinnacles National Park. There are also two release sites outside of the study area: one in Baja California, and one in northern Arizona. A new release site, which is the focus of this study, has been proposed for northern California, near the Oregon border (see Fig 1).

Major mountain ranges are oriented along a north-south axis and include the Coast Ranges, the Cascade Mountains, and the Sierra Nevada. Wide valleys, including the Central Valley in California and the Willamette Valley in Oregon, separate the Coast Ranges from interior mountain ranges. The Tehachapi Mountains in southern California and the Klamath and Siskiyou Mountains in northern California and southern Oregon connect the inland and coastal mountain ranges. The climate ranges from Mediterranean in southern and central coastal California to temperate rainforest in coastal areas of northern California, Oregon, and Washington. Precipitation and temperature are highly variable along elevation and latitudinal gradients in interior portions of the study area, resulting in large variation in ecological communities.

### Landscape conductance surface

We developed a map of relative landscape conductance for California condors in flight by modeling the relationship of environmental covariates to known locations of condors aloft. Specifically, we used MaxEnt version 3.3.3k, a maximum entropy–based machine learning computer program that estimates the probability distribution of a species’ occurrence based on a given set of environmental constraints [22–24]. Presence-only models, such as MaxEnt, are preferred over presence/absence models in nonequilibrium situations, including when modeling expanding populations of imperiled species [25]. MaxEnt models can be conservatively interpreted as a relative index of environmental suitability, where higher index values depict better conditions for the species [22]; in this case, better conditions for the occurrence of condors in flight. We focused our analysis at the landscape-scale with a 1 km^2^ spatial grain given that our ultimate goal was to identify landscape-scale connectivity and movement probabilities. We trained and tested models using data from the background area (see Fig 1) but projected them to the entire study area. We built simple models given the large spatial extent and coarse-resolution of the spatial data and to avoid overfitting the model to the training region, which would likely degrade its performance when we project it to novel geographic areas [26].

#### Condor occurrence data

We downloaded, from Movebank (www.movebank.com), raw condor locations of all California condors equipped with Global System for Mobile Communications (GSM)/Global Positioning System (GPS) transmitters in the southern and central California flocks. This included occurrence data spanning July 2013-May 2017 for 75 individual condors: 29 from central California release sites (1,939,737 GPS points), and 46 from the southern California release site (3,453,959 GPS points) (Fig 1). The occurrence data included juveniles and adults (breeding and non-breeding) of both sexes, as well as captive-reared and wild-fledged individuals (S1 Table). GSM/GPS transmitters were patagial-mounted and deployed on a subset of the condor population during routine handling [27]. GSM/GPS transmitters record location, altitude, and accuracy data every 2–30 minutes and transmit these data to a GSM network or Code-Division Multiple Access (CDMA) network. We filtered raw GPS data using the following procedure:

1. We selected only those points with reliable GPS fixes (“proofed” = 1) that likely represented condors in flight (“speed” > 2.78 meters per second (m/s) and < 30.0 m/s). Below we refer to our filtered data as movement points because we based all of our analyses on occurrence points from condors in flight.
2. We removed points that were located offshore. We also removed points within 5 km of existing release sites and the Hopper Mountain National Wildlife Refuge (NWR) flight pen (a former release site which is occasionally used to trap condors) because the frequency of condor movement within these areas is artificially inflated by periodic baiting to recapture condors for health screenings and to replace transmitters. Free-flying condors are also attracted to these sites when captive condors are held in the flight pens prior to release. We chose 5 km based on inspection of frequency plots, which showed a marked decline in the number of movement points at this distance.
3. MaxEnt requires that occurrence points are independent, as autocorrelation can lead to a biased model. We can minimize bias from autocorrelated GPS locations by rarifying data at regular time intervals over a relatively long period [28, 29]. Therefore, we further filtered our occurrence dataset to include only one randomly selected movement point per individual condor, per day, for all days that GPS positions were available. This resulted in 22,251 filtered points for southern California and 6,839 filtered points for central California (S1 Table). Given the multi-season and multi-year dataset, as well as the ability of individuals to move long distances, condors in our study had the potential to access various landscapes throughout the annual cycle. Thus, occurrence datasets were sufficient to describe the landscape-scale ecological relationship between condors in flight and environmental covariates.

#### Background data

MaxEnt requires that the user specify a background (i.e., area available for the species to select) against which one compares covariate values at occurrence points [22, 24]. We created MaxEnt models within the environmental and geographic space of the background area, but projected models across the entire study area. Choice of an appropriate background sample is crucial to avoid sample selection bias and, consequently, poor performing models [30]. We generated a background area by defining a convex hull around all condor movement points (see **Condor Occurrence Data**, above), buffering the resulting polygon by 10 km to account for the area outside of the strict hull boundary that was presumably available for condors to select, and clipping the polygon to the coastline. We then produced 10,000 random points within the buffered convex hull to serve as background data (see Fig 1). We used 10,000 points, as previous work has shown this is sufficient to represent the range of environmental conditions across large study areas [31]. To match the condor occurrence dataset, we excluded background points from the area within 5 km of each release site and the Hopper Mountain NWR flight pen.

#### Environmental covariates

As obligate soaring birds, California condors rely on upward air movement for staying aloft [32, 33]. When lift is favorable due to winds deflecting off mountains, or due to thermal updrafts, condors can move long distances expending limited energy. From an evolutionary perspective, this ability for obligate scavengers to move long distances with minimal energy expenditure is a critical adaptation to finding carrion—an ephemeral resource [34, 35]. Therefore, we hypothesized that upward air movement influences landscape conductance for condors. We also hypothesized that condor movements are constrained by the distribution of terrestrial habitats (e.g., areas of low percent tree canopy cover, low human disturbance, high primary productivity, rugged terrain, and steep slopes).

In developing our MaxEnt models, we considered seven covariates related to thermal updrafts, terrain (a proxy for orographic lift), terrestrial condor habitat, and human disturbance (Table 1). We selected covariates based on published species-habitat associations [17, 33, 36–38], species-habitat models developed for other vultures [39–43], and the availability of GIS data at the appropriate spatial scale spanning the entire study area. For covariates we suspected to be correlated with landscape-scale habitat selection (i.e., at a larger scale than our spatial grain of 1 km^2^) we summarized focal statistics within a 10 km neighborhood (Table 1). We used a 10 km neighborhood recognizing that large vultures in-flight have the visual acuity to perceive landscape features and resources (at a resolution as small as 2 m) at distances of up to 10 km [44, 45].

**Table 1:** Covariates used in developing a California condor landscape conductance surface.

Covariate	Description	Data Source
Thermal Updraft Velocity	Annual mean velocity of rising air (m/s)	Regional Atmospheric Soaring Prediction Maps (http://www.drjack.info/RASP/index.html)
Terrain Ruggedness	Ratio of 3-dimentional surface area to planar surface area within a 10 km neighborhood	The National Map Small-Scale Collection (https://nationalmap.gov/small_scale/mld/elev100.html) processed with DEM Surface Tools for ArcGIS 10 (http://www.jennessent.com/arcgis/surface_area.htm)
Slope	Mean slope (degree)	The National Map Small-Scale Collection (https://nationalmap.gov/small_scale/mld/elev100.html)
Tree Canopy Cover	Median tree canopy cover (%)	National Land Cover Database 2011 (http://www.mrlc.gov/nlcd2011.php)
Terrestrial Habitat	Density of terrestrial habitat within a 10km neighborhood	Derived from California condor nesting and feeding models [17]. We used focal statistics in ArcGIS 10 to calculate the mean amount (km^2^) of terrestrial habitat (presence of either nesting or feeding habitat) within 10km of each cell.
Road Density	meters of road/km^2^ within a 10km neighborhood	Data Basin (http://databasin.org/datasets)
Human Population Density	humans/km^2^ within a 10 km neighborhood	2010 TIGER/Line Census Data (https://www.census.gov/geo/maps-data/data/tiger-data.html)

To identify correlated covariates, which might reduce model interpretability, we assessed multicollinearity among covariates by calculating univariate pairwise Spearman correlation coefficients (*r*_*s*_) based on values of each variable at condor movement points in each modeling region. If two covariates had *r*_*s*_ > 0.70, we retained only one of the pair to aid in interpretation of model results, retaining the covariate that had greater biological meaning.

#### Model selection and settings

We developed several *a priori* hypotheses describing the relationship between condor movement and covariates based on our knowledge of obligate soaring birds, and condors specifically, and created MaxEnt models for each hypothesis (Table 2). We evaluated our models using 5-fold cross-validation. We calculated several measures of model performance on the data withheld for testing for each fold, including mean values of regularized training Gain, test AUC, and AIC [22, 46]. We then averaged these measures across folds. We selected the model with the lowest mean AIC as the best performing model that we then projected to the remainder of the study area. We used the logistic output of this projection as a measure of relative landscape conductance for California condors. We considered whether non-linear transformations of our species distribution model output might produce more accurate conductance surface [47]; but, we retained a simple linear relationship because our occurrence data consisted of condors that were in-flight and moving.

**Table 2:** Mean relative contribution of covariates (%), and measures of model performance for a California condor landscape conductance surface (Some columns in the upper portion of the table do not sum to 100 due to rounding).

Covariate	Model 1	Model 2	Model 3	Model 4	Model 5
Slope	9.8	10.1	10.0	92.0	100
Terrain Ruggedness	0.2	0	0.1	0.1	0
Thermal updraft velocity	0.5	0.5	--	8.0	--
Tree canopy cover	0.8	0.7	0.8	--	--
Modeled terrestrial habitat	88.6	88.6	89.1	--	--
Human population density	0	--	--	--	--
Road density	0	--	--	--	--
**Model Performance Measures**					
AIC	125413	125418	125440	148034	152022
Regularized Training Gain	1.48	1.48	1.47	0.68	0.60
Test AUC	0.91	0.91	0.91	0.81	0.80

For model settings, we used only linear and quadratic features, set the maximum number of model iterations to 5,000, implemented clamping, and did not remove duplicate presence records or add occurrence data to the background.

### Circuit theory and condor movement

We defined three modeling regions to predict condor movements, centered on existing or proposed release sites: (1) southern California, with an existing release site at Bitter Creek NWR, (2) central California, with existing release sites at Pinnacles National Park, the Santa Lucia Mountains adjacent to Big Sur, and the mountains near San Simeon, and (3) northern California, with a proposed release site in the Bald Hills of Redwood National Park (Fig 1). Modeling regions included all areas within 350 km of the above release sites. We chose 350 km to encompass the farthest Euclidean distance of condors from release sites in our occurrence data. This distance was uncorrected for topography because condors in flight can skirt topographic relief.

Within each modeling region, we used Circuitscape software v 4.0 to model California condor movement through the landscape [48]. Circuitscape uses algorithms from electrical circuit theory to model connectivity, treating landscapes as resistance (or conductance) surfaces where one can model patterns of animal movement as electrical current between sources and destinations across a resistor [18]. When one considers combinations of sources and destinations, the result is a continuous map of electrical current and voltage across all possible routes in the network [18].

Electrical current in Circuitscape is ecologically interpreted as the net probability of movement between a source and a destination through a resistance or conductance surface [18]. The conductance surface for our analyses was the logistic output of the MaxEnt model described above. We defined the source of electrical current as points centered on existing release sites within each modeling region. Given our lack of knowledge about the destination of condors, we generated points representing ground nodes at 10 km intervals along the edges of our modeling regions (i.e., along a 350-km buffer that encircled the respective release sites). We placed ground nodes in all directions away from the release sites to avoid directionality bias. Our ground nodes extended beyond our study area and landscape conductance values, meaning we needed to assign conductance values to grid cells outside of the study area, including the Pacific Ocean, Nevada, and Baja California. Large vultures avoid, or have difficulty, crossing large bodies of water [49–51]; therefore, we assigned the minimum conductance value observed in our study area to the Pacific Ocean. Given the unknown conductance in terrestrial areas in Nevada and Baja, we assigned all cells in these areas the median conductance value observed in our study area; we used median values to provide a constant surface for electrical flow outside of the study area rather than for estimating net movement probabilities in these areas.

We ran Circuitscape in Advanced Mode, iteratively connecting each source node to each ground node within each modeling region. This allowed us to model condor net movement probabilities in all directions away from release sites across the network, creating individual current maps for each source-ground node connection in the network for each of the three modeling regions. For each modeling region, the mean of the individual current maps provided a direction-neutral connectivity map. The default units of electrical current maps were amperes (A), which we converted to milliamps (mA) by multiplying outputs by 1,000 so we could work with simpler numbers. To assess the relationship of electrical current to the prevalence of condors in flight, we extracted the electrical current values to condor movement points and plotted the reverse cumulative frequency of these values in 1 mA intervals. We used a reverse cumulative frequency plot, as we were interested in how the frequency of condor movements might attenuate in relation to electrical current.

We assessed the degree to which mean electrical current related to the predicted/expected ratio of condors moving after their release (from release sites, which we treated as sources of electrical current) using the continuous Boyce Index and Predicted/Expected (P/E) curves produced by the ECOSPAT package in R [52, 53]. Given known biases around ground nodes [54], we limited our evaluation of the models to within 300 km from release sites. The continuous Boyce index and continuous P/E curves calculated in ECOSPAT are presence-only and threshold-independent measures that provide information on model resolution and predictive performance across the spectrum of habitat suitability values (or, in our case, electrical current values) within each modeling region [52, 53]. Boyce index values vary from -1 to +1, with values closest to +1 indicating a model where predictions were consistent with the distribution of the movement points. Values close to zero indicate that the model is not different from a random model, and negative values indicate an incorrect model. An ideal model would have a P/E curve that was linear and positive, where electrical current was proportional to the probability of use by released condors in flight.

### Landscape connectivity of reintroduction sites

We evaluated landscape connectivity for California condors between the proposed release site and existing release sites in California using Linkage Mapper v. 2.0.0 [55]. Linkage Mapper uses a landscape resistance surface and a map of core areas to identify least-cost paths between core areas [55]. Our resistance surface was the inverse of the conductance surface described above (1 /Conductance) [18]. We defined core areas as generalized nesting habitat patches by reclassifying D’Elia et al’s [17] logistic map of relative nesting habitat suitability into a binary output using a threshold value of 0.04 [17], and removing all patches smaller than 10km^2^. We then built network and map linkages using the cost-weighted network adjacency method in Linkage Mapper to identify core areas to connect, dropping corridors that intersected with core areas. Linkages were visualized using cost-weighted distances, where higher values represent weaker connectivity among modeled nesting habitat patches.

## Results

### Landscape conductance surface

Our habitat suitability model of condors in flight generally aligned with the current and recent historical distribution of the species. Given the high relative contribution of terrestrial habitat to our in-flight model (see Table 2), factors that most influenced that model (i.e., areas of high topographic relief, low percentage of tree canopy cover, and high landscape productivity [17]) were most favorable to condor movements and therefore more likely to be occupied by condors in flight. Our model predicted that areas over deserts, dense forests, and wide flat valleys were less likely to be occupied by condors in flight. Correlation coefficients of univariate pairs of environmental covariates were all below 0.70; therefore, no covariates were removed prior to development of an in-flight condor habitat model. The model with the lowest AIC, and therefore the one that we selected to represent our conductance surface, was the model that included all covariates (Table 2).

### Circuit theory and condor movement

**Fig 2:**
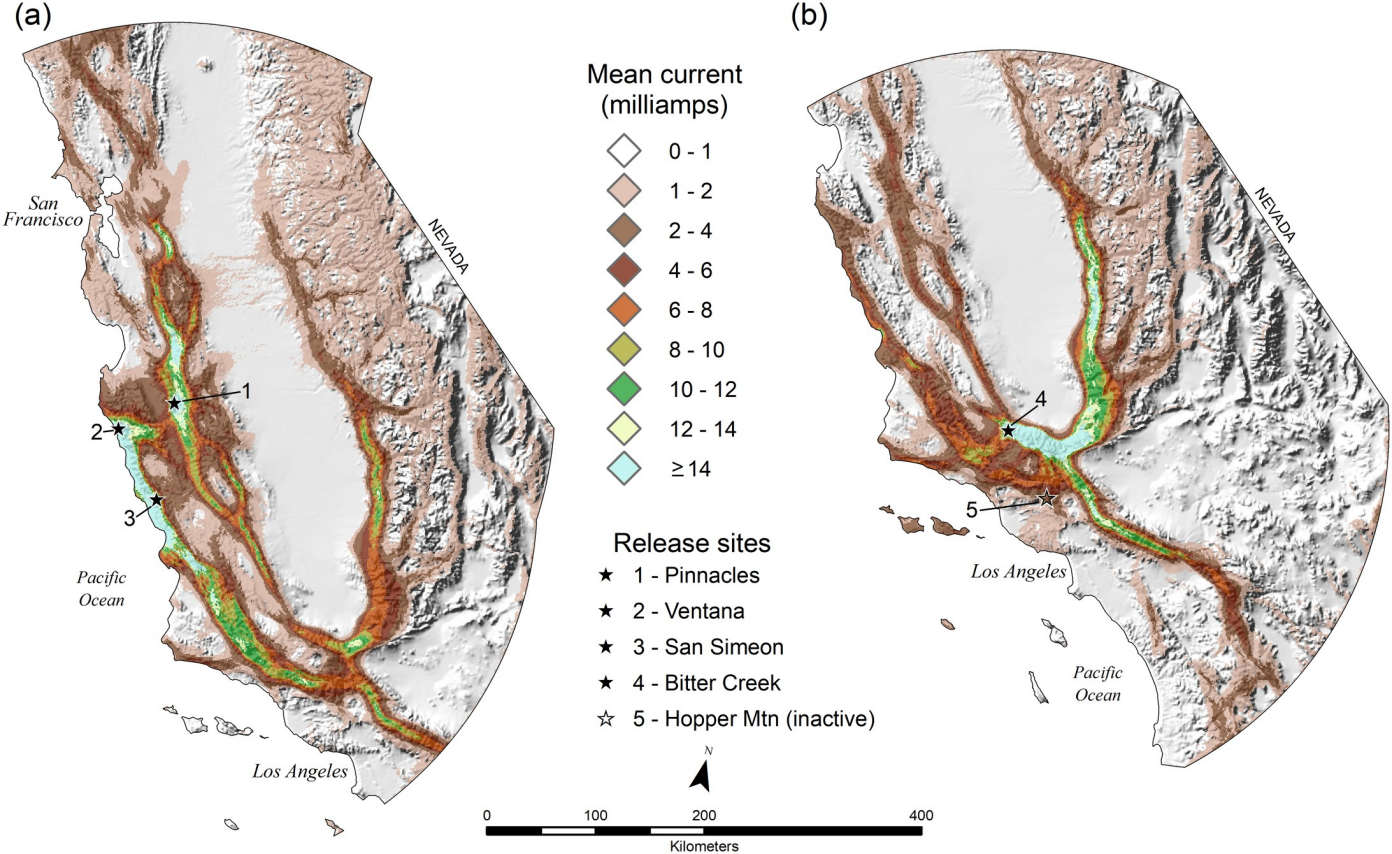
Mean electrical current for California condor circuit theory models in (a) central California and (b) southern California, USA.

**Fig 3:**
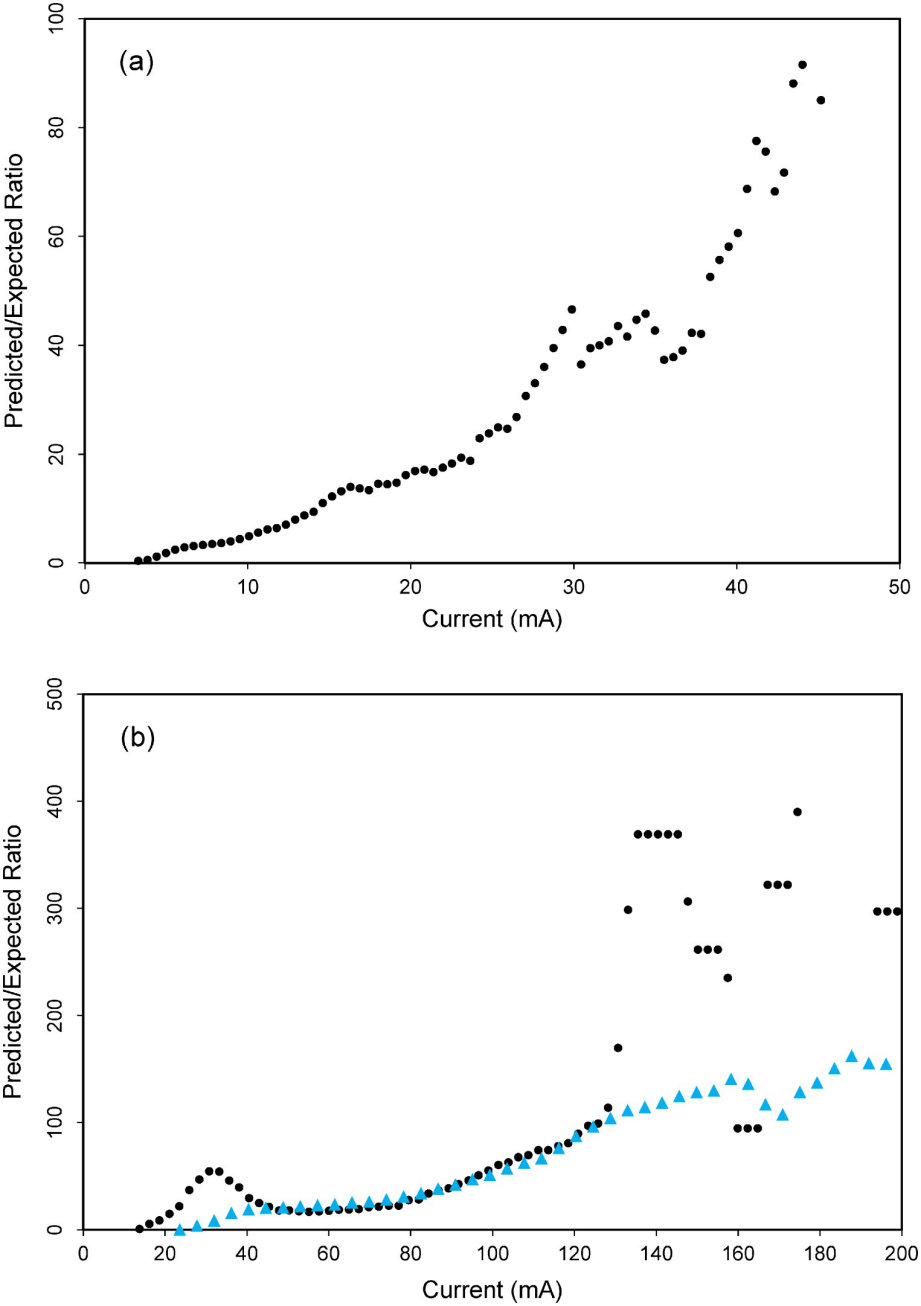
Continuous predicted/expected plots of electrical current models for California condors in (a) central California and (b) southern California, USA. Blue triangles in (b) represent the predicted/expected plot when Hopper Mountain National Wildlife Refuge and the Tehachapi Mountains were added as additional ground nodes in the electrical circuit.

Mean electrical current generated via Circuitscape (1,166 source-ground connections, 220 for southern California, 726 in central California, and 220 in northern California) was well calibrated with the known pattern of condor movement in southern and central California (Figs 2 and 3). The continuous Boyce Index for electrical current in southern California was 0.86 and in central California was 0.98 (Fig 3). A spike in the P/E plots, between approximately 20 and 40 mA in southern California, corresponds with a higher than expected density of condor movement points in the Tehachapi Mountains. We suspected this was because Hopper Mountain NWR, an area where condor nesting activity is concentrated and where baiting and trapping occurs—had an extremely high frequency of condor movement that was unaccounted for in our model. This prompted us to conduct a *post-hoc* analysis to determine whether adding prior information on destination nodes within the circuit—representing condors moving from release sites to areas of high use at Bitter Creek NWR, the Tehachapi Mountains, and Hopper Mountain NWR—would improve model fit. Specifically, we used Circuitscape to develop a new current map based on pairwise connections between the release site (i.e., the source node) and the center of two high-use areas noted above (i.e., ground nodes). We then overlaid the new map with the original map of electrical current and took the maximum value of the two surfaces. We found that accounting for movement to these high-use areas via our *post-hoc* analysis, improved the continuous Boyce Index from 0.86 to 0.95, and smoothed out the spike in the P/E curve (Fig 3).

**Fig 4:**
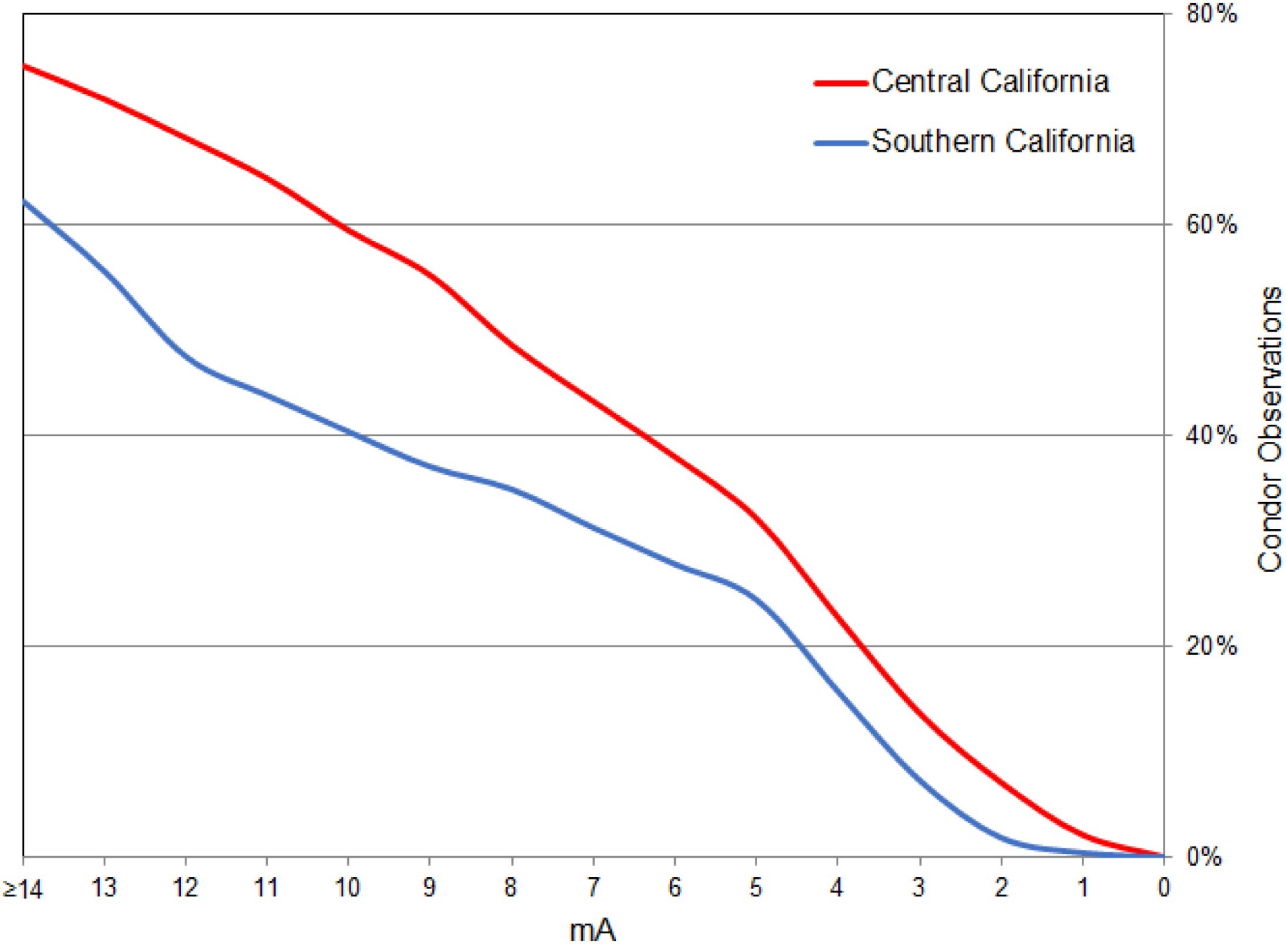
Reverse cumulative frequency plot of in-flight California condor locations (July 2013-May 2017) in relation to electrical current for the southern and central California modeling regions, USA.

**Fig 5:**
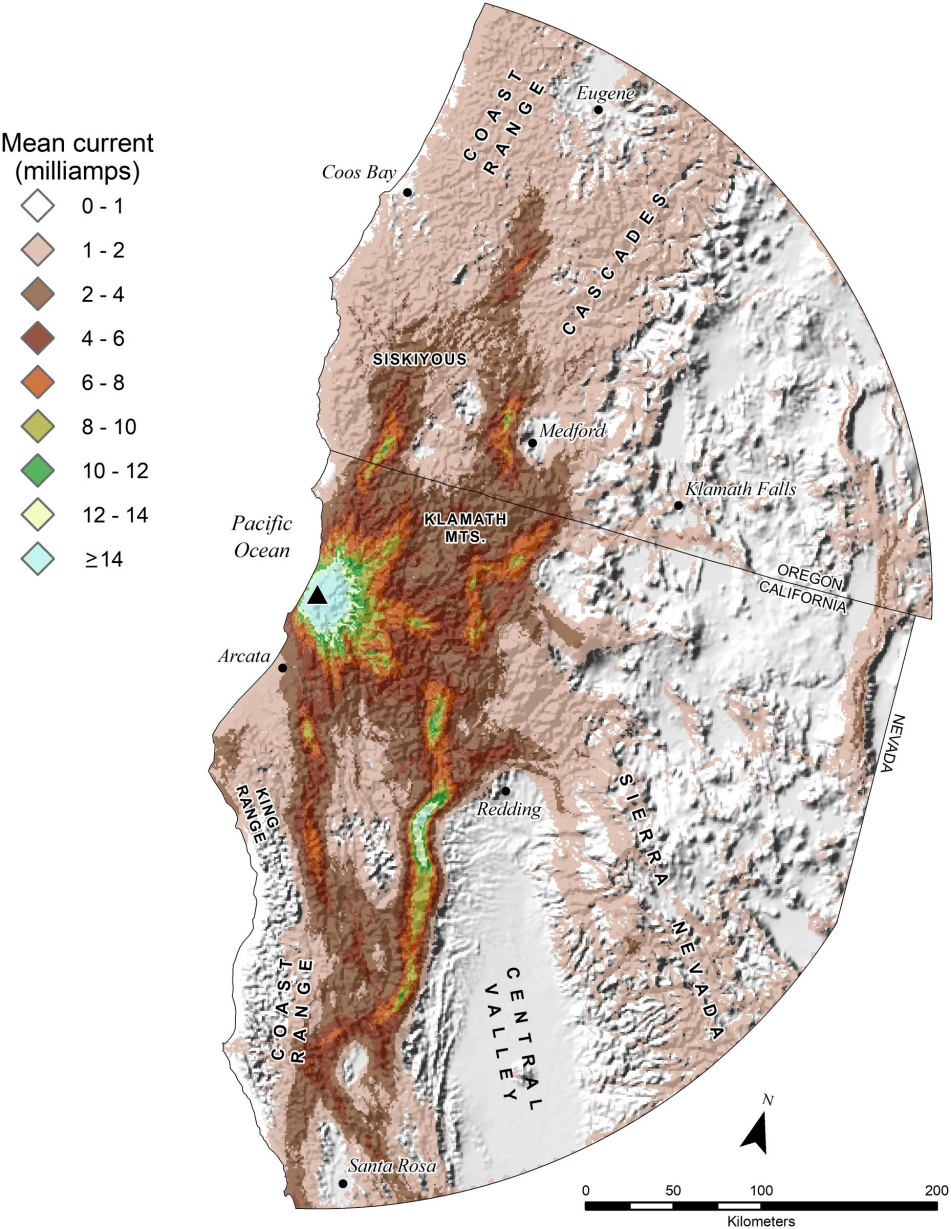
Mean electrical current for a California condor circuit theory model around a proposed release site (▲) in northern California, USA.

The number of condor movement points attenuated away from release sites, concordant with the attenuation of electrical current (Fig 4). Reverse cumulative frequency plots of condor movement declined roughly linearly, with approximately 93–98 percent of movement points contained within areas ≥2 mA (Fig 4). In northern California, our model of electrical current predicted that condor movements would likely be initially most concentrated in northwestern California and southwest Oregon (Fig 5). Specifically, areas likely to encompass >90 percent of condor movements were generally concentrated in the Klamath, Siskiyou and North Coast Ranges (including the King Range), extending south to Bodega Bay, inland to the Mt. Shasta region, and north to the Umpqua Valley.

### Landscape connectivity of reintroduction sites

**Fig 6:**
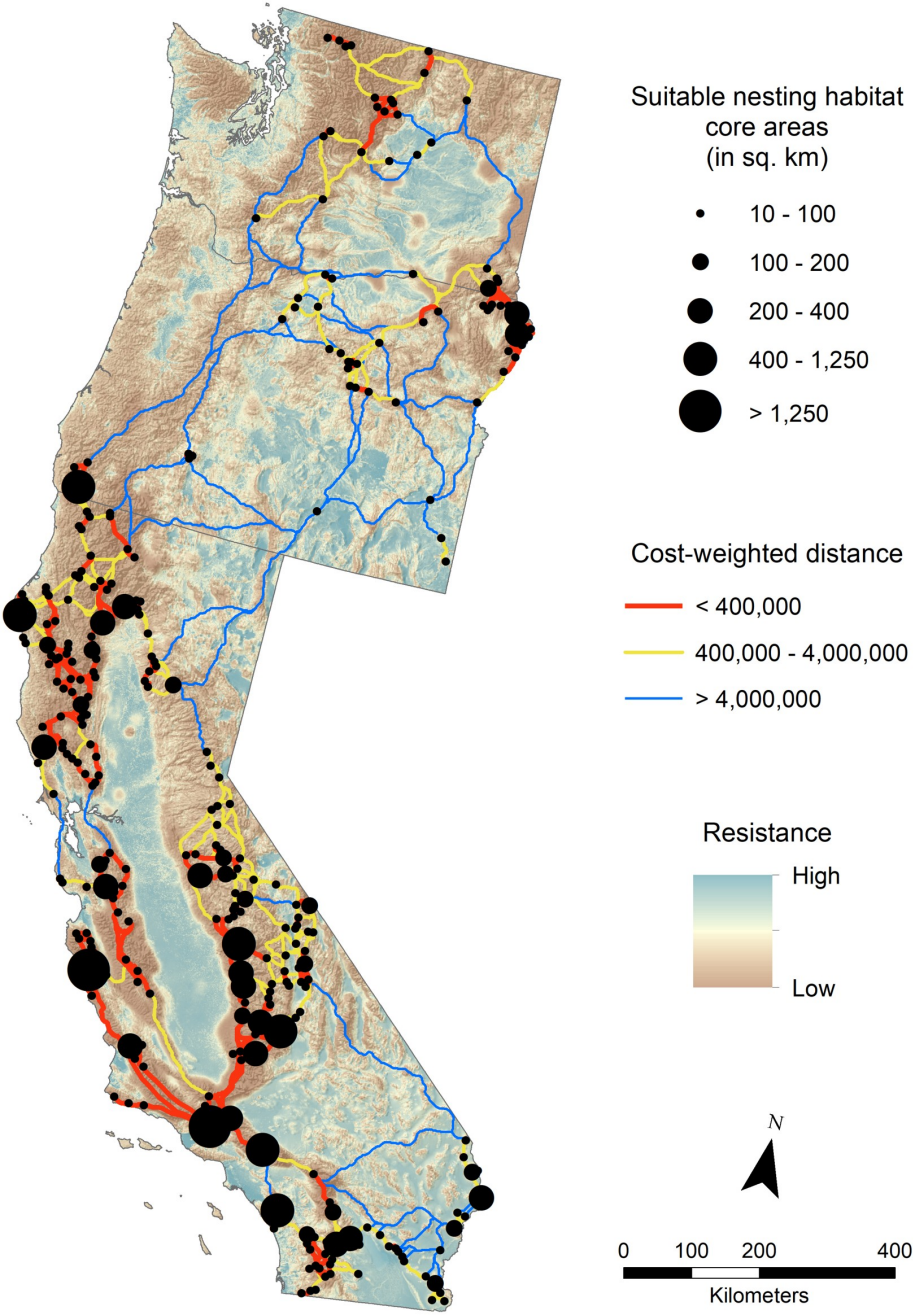
Least-cost-path linkages between California condor modeled core nesting habitats (≥10 km^2^) in Washington, Oregon, and California, USA. For display purposes, core nesting habitats are represented as points and linkages between large core nesting habitats were snapped to points through core nesting habitats while maintaining their least-cost path distances.

Our cost-weighted distance linkage network provided a useful visualization of areas of high and low connectivity among modeled nesting habitat patches. Our linkage network consisted of 305 modeled nesting habitat patches that met the minimum size criteria of 10 km^2^ (Fig 6). Linkage mapper produced a network of 553 least-cost-path links between nesting habitat patches, with a mean cost-weighted distance of 177.6 (in cost units, not geographic distance). Areas of high cost-weighted distance between core nesting areas near the proposed release site and the existing release sites included the San Francisco Bay area (mean cost-weighted distance among two links = 753.5) and the northern Sierra Nevada mountains (cost-weighted distance = 428.3). We found an even higher cost-weighted distance between core nesting areas near the proposed release site in southwest Oregon and core nesting areas in northern and eastern Oregon (mean cost-weighted distance > 1,000).

## Discussion

### Landscape conductance surface

As we predicted, our models indicated that condors showed an affinity for moving over terrestrial nesting or feeding habitats and over areas with moderate to steep slopes. They also showed strong avoidance of wide, flat valleys and areas of low primary productivity where ungulate densities were relatively low. This makes ecological sense, as we would expect condors in flight to spend most of their time searching for food in areas of slope-lift in open or semi-open habitats, patrolling nesting territories, or commuting between nesting and feeding areas. It is also intuitive that condors would be less likely to travel across expansive flat areas where they risk being grounded, or through areas where food densities are low. We recognize the possibility that some valleys may become accessible to condors when summer thermals provide sufficient lift for sustained soaring flight. It is also possible that some areas with low annual average productivity may occasionally be attractive to condors due to seasonal movements of native ungulates or livestock.

Contrary to our expectations, annual thermal updraft velocities appeared relatively unimportant to the relative frequency of moving condors. This may partially be a function of our use of a covariate for thermal lift that is based on average values across the annual cycle. Others have found significant relationships between meteorological variables and condor space use at finer temporal scales [33, 38], suggesting that landscape resistance to condor movements is dynamic, and meteorological “gates” between different parts of the landscape likely emerge and vanish throughout the year. Seasonal models using the techniques we describe here could help elucidate these temporal changes in condor space-use across the annual cycle.

We predicted significant areas of suitable in-flight condor habitat around the proposed northern California release site. It is important to emphasize that our predictions are based on a species distribution model that is inherently correlative, rather than process-based [56]; and therefore, represents a working hypothesis based on data from other regions. Several studies have highlighted the potential hazards of projecting species distribution models into novel areas [57–60]. Yet, when constructed with careful consideration of a species’ ecology, distribution models remain a transparent, quantifiable, and repeatable method for developing and testing hypotheses about potential habitat in occupied regions. Projecting models to novel areas can be reliable enough for effective decision-making provided that the data and models are reasonable and any correlations across covariates are stable through the temporal and geographic domains for which the predictions are made [58, 61]. Falsification or support for our hypotheses will be possible through evaluating whether our models are correlated with the distribution of condors once a population is established in northern California.

We limited our analysis to a small subset of ecologically relevant covariates and smooth response curves, as is recommended for large spatial extents [26, 62]. Thus, our results represent relative habitat suitability at the landscape scale and were not intended to elucidate finer-scale habitat selection. Future efforts to develop landscape conductance surfaces could explore this finer-scale habitat selection by: (1) using a hierarchical approach [63] with a smaller spatial grain; (2) including environmental covariates on seasonal or real-time wind direction and speed in relation to topographic features; (3) including seasonal or real-time meteorological conditions; and, (4) including direct measures of terrestrial and marine food availability. In addition, separating occurrence points into categories of movement (e.g., linear soaring, gliding, and circular soaring), or specific parts of a species’ life cycle [64], might also improve our ability to make finer-scale predictions [51, 65–67].

Species distribution models are grounded in the ecological theory that species distributions are determined, at least in part, by environmental covariates and that reasonable approximations for these covariates can be estimated [62]. Therefore, it is important to acknowledge potential weaknesses in the species-environmental covariate relationship when interpreting species distribution model outputs. Our covariate for terrestrial habitat, which had the highest percent relative contribution to our conductance surface, likely underestimated feeding habitats along the coast for three reasons: (1) our model of feeding habitat was based on a higher proportion of observed inland feeding occurrences than coastal feeding occurrences, (2) the coastal feeding occurrences were limited to a small section of coastline near the Big Sur reintroduction site and may not represent the full range of suitable coastal feeding conditions, and (3) we did not include direct or indirect covariates representing coastal food availability (e.g., distribution of marine mammal haul-outs or marine mammal strandings) or on-shore winds [17]. Thus, relative habitat suitability and conductance values along the immediate coast are likely biased low and should be viewed with caution. Our experience suggests that coastal areas with relatively consistent diurnal onshore winds and steep slopes are likely to be favorable to condor movement and may be an important corridor for north-south travel, at least in certain seasons. In addition, use of sea breezes deflecting off terrain or sea breeze fronts has been documented in other large soaring birds [49, 68, 69].

Our terrestrial habitat covariate likely underestimated nesting habitat in forests given the higher prevalence of cliff and cave nesting condors in our dataset, and our covariates did not include direct measures to predict the distribution of potential nesting trees. Thus, areas of the northern California coast along the redwood belt, with concentrations of large redwood trees suitable for nesting, and areas of the Sierra Nevada with groves of giant Sequoias or other nesting habitat, may be more connected than depicted in our models. Studies of other large soaring vultures suggest they are more frequently observed in open or semi-open habitats than in densely forested habitats while foraging [29, 70, 71]. This is likely due to their reliance on vision for finding food, rather than olfaction [72]. Whereas areas of dense trees may not be used by condors in southern and central California in greater proportion to their availability [38], this pattern may not hold as the distribution of condors expands into areas of the historical range where nest trees are more widely available, or where nesting and foraging areas are separated by forests (see [73]).

The redwood coast has an exotic humid climate and is perhaps the most famous example of a cloud-connected coastal ecosystem for its high incidence of summertime fog [74]. We considered including a covariate that accounted for fog in our in-flight condor models, expecting that areas of extensive and frequent fog might inhibit condor movements due to decreased visibility. However, data limitations precluded evaluation of seasonal differences in fog frequency, duration, or intensity. If finer scale datasets become available, we recommend including seasonal fog characteristics for future modeling efforts, as we suspect it may play a role in space use at these finer scales. Daily and hourly changes in wind patterns due to temperature gradients and terrain (e.g., surface wind, sea breeze, slope wind, valley wind, and mountain wind; see [75, 76]) also may be important in understanding finer-scale landscape conductance; therefore, their inclusion in finer-scale species distribution models for the California condor should be considered.

### Circuit theory and condor movement

We found that electrical current was correlated with condor movement around existing reintroduction sites. Our models were able to discriminate between areas condors were likely to move or avoid because of the great variation in environmental covariates that had significant relationships to landscape conductance in our modeling regions. For species where landscape conductance around the release site lacks significant variation, circuit theory models may offer little advantage over simple Euclidean distances and focusing on finer-scale habitat attributes may be necessary to understand these colonization patterns [20].

We developed simple electric circuits where the only source of individuals was at the release sites and we systematically placed grounds around the release sites assuming no directional bias. However, we recognize that there may be opportunities to improve our model of net movement probabilities by incorporating additional electrical sources and grounds of specific “strengths” (amps and resistances, respectively; see [48]). For example, in addition to reintroduction sites, one could specify nesting areas likely to be used by the reintroduced population as additional potential sources of individuals, with the strength of the source scaled relative to the productivity of the nesting habitat. It may also be prudent to specify source-node strengths for individual release sites depending on the number of animals released or the amount of time that the release site has been in operation—factors that are highly correlated for condors [77]. If information on likely destinations (e.g., important feeding areas or overnight roosting sites) were available, one could place grounds with specific resistance values within the modeling regions to improve upon our directionally naïve models. Adding sources and destinations may be important when breeding grounds are concentrated or where there are other attractants that affect a large proportion of the population. For example, our model in southern California underpredicted net movement probabilities around Hopper Mountain NWR and in the Tehachapi Mountains. We were able to significantly improve model performance by including additional ground nodes in these areas. While increasing model complexity can come at a cost of less generalization to novel environments, it may be useful for modelers to experiment with various configurations and strengths of possible attractants within their modeling region to assess the potential impact of these sources and attractants on predicted movement probabilities of the translocated species.

Unexplored in our analyses are the location of nearby release sites and the role of social tradition or social attraction in condor movements (see [78, 79]). Our anecdotal observations suggest that opening a new release site within commuting distance of an existing release site is a significant attractant to California condors. For example, when condors were first released at San Simeon in 2015, multiple adults from Big Sur began frequenting the San Simeon area almost immediately. Two individuals from Big Sur nested in the vicinity of the new release site shortly thereafter—something reintroduced condors had not done previously despite approximately 20 years of reintroductions at Big Sur [80, 81]. Social attraction from establishment of new release sites has also been observed in condors in southern California and in other vulture reintroduction programs in Europe [82, 83]. In addition, social attraction may play a role in the expansion of vulture populations as individuals discover new nesting or feeding areas. While our models focus on the relationship of condor movements to landscape features, they do not account for social tradition or social attraction. Future efforts to account for these factors may improve predictive capabilities.

More sophisticated analytical and simulation models are available that can produce results similar to, or in some cases superior to, those produced by circuit theory [18]. Unlike individual-based movement models and agent-based models (e.g., [84]), circuit theory models do not depict individual selection of locations during movement or exploration *per se* (see [85]) and do not account for intra- or interspecific competition [86], settlement patterns [87, 88], temporal scale, Allee effects [89], or sex- or age-specific habitat use, selection, or movement. Despite these shortcomings, they are relatively easy to construct, require minimal parameterization, and can be useful on their own or as a building block for individual-based simulations. Thus, circuit theory models may be an effective intermediate step prior to the more involved modeling of individual movements [18] and can provide insights into the appropriateness of a resistance/conductance surface prior to its use in an individual-based model. Further, individual-based models typically require data on movement step distance, turning angles, and related motion parameters to vary by habitat conditions and by sex and age class of the individual (e.g., [90, 91]). Circuitscape requires essentially none of this, while still providing a reasonable approximation of how animals may radiate from a translocation site.

### Landscape connectivity of reintroduction sites

Identifying how, and to what extent, a translocated population of animals might be ecologically connected or disconnected with existing populations is of central importance in planning reintroductions and conservation translocations. In most cases, there is an explicit goal of connecting populations to form or maintain a metapopulation with adequate gene flow (e.g., [92, 93]). However, for some species there are legal reasons for having initial separation between translocated populations and existing populations—for example, if a threatened or endangered species is reintroduced as an experimental population under the ESA [94]. There may also be a desire to keep populations separate to reduce disease transmission risk [95], or when the genetic risks of connectivity outweigh the benefits [96].

Our habitat linkage maps for condors generally agree with the existing and historical distribution of condors and the anticipated movement pathways of condors that follow large north-south mountain ranges in California. They are also concordant with the available genetic information from the historical population, which did not reveal any geographically isolated mtDNA haplotypes from the Pacific Northwest to Baja California, Mexico [97]. High cost-weighted distance between modeled nesting habitats across the San Francisco Bay area and across the forests of the northern Sierra Nevada indicates relatively high landscape resistance to condor movement between these areas. However, given the distances condors can travel [27, 98], it is unlikely these areas will significantly restrict gene flow once a population becomes established in the Pacific Northwest. Our linkage model indicates that connectivity between core nesting areas around the proposed release site is most restricted to the north and east. However, we need more data on nesting habitats and movement patterns in dense forests before drawing reliable conclusions regarding landscape connectivity through the forested regions of northern California, Oregon, and Washington [17]. We emphasize that habitat and population linkages are dynamic, and that further work to evaluate seasonal changes in habitat and population connectivity are likely to reveal changes across the annual cycle that are not evident in our predictions that rely on annual averages.

Conservation translocations are adaptive challenges in that they are complex and require solutions that blend technical expertise with social values [99]. Managers need objective predictions of where animals are likely to move once released to effectively communicate with stakeholders and to proactively mitigate threats. Our case study of California condors shows that circuit-based models are a potential useful tool for managers to consider when planning conservation translocations.

## Supporting information

S1 Table California condors outfitted with Global System for Mobile Communications transmitters or GPS transmitters from July 2013 to May 2017, California, USA. (DOCX)

## References

[1] Seddon PJ, Armstrong DP, Maloney RF. Developing the science of reintroduction biology. Conservation Biology. 2007;21:303–12. 10.1111/j.1523-1739.2006.00627.x 17391180

[2] Brichieri-Colombi TA, Moehrenschlager A. Alignment of threat, effort, and perceived success in North American conservation translocations. Conservation Biology. 2016;30:1159–72. 10.1111/cobi.12743 27119768

[3] Griffith B, Scott JM, Carpenter JW, Reed C. Translocation as a species conservation tool: status and strategy. Science. 1989;245:477–80. 10.1126/science.245.4917.477 17750257

[4] Letty J, Marchandeau S, Aubineau J. Problems encountered by individuals in animal translocations: lessons from field studies. Ecoscience. 2007;14:420–31.

[5] Dunham JB, White R, Allen CS, Marcot BG, Shively D. The reintroduction landscape: finding success at the intersection of ecological, social, and institutional dimensions In: Jachowski DS, Millspaugh JJ, Angermeier PL, Slotow R, editors. Reintroduction of fish and wildlife populations. Berkeley, California: University of California Press; 2016 p. 79–103.

[6] Torres RT, Carvalho J, Serrano E, Helmer W, Acevedo P, Fonseca C. Favourableness and connectivity of a Western Iberian landscape for the reintroduction of the iconic Iberian ibex *Capra pyrenaica*. Oryx. 2017;51:709–17.

[7] Bar-David S, Saltz D, Dayan T. Predicting the spatial dynamics of a reintroduced population: the Persian fallow deer. Ecological Applications. 2005;15:1833–46.

[8] Bar-David S, Saltz D, Dayan T, Shkedy Y. Using spatially expanding populations as a tool for evaluating landscape planning: the reintroduced Persian fallow deer as a case study. Journal for Nature Conservation. 2008;16:164–74.

[9] Kuemmerle T, Perzanowski K, Akcakaya HR, Beaudry F, Van Deelen TR, Parnikoza I, et al Cost-effectiveness of strategies to establish a European bison metapopulation in the Carpathians. Journal of Applied Ecology. 2011;48:317–29.

[10] Carroll C, Phillips MK, Lopez-Gonzalez CA, Schumaker NH. Defining recovery goals and strategies for endangered species: The wolf as a case study. BioScience. 2006;56:25–37.

[11] Snyder NFR, Snyder HA. The California Condor: A Saga of Natural History and Conservation. San Diego, CA: Academic Press; 2000.

[12] Mee A, Hall LS. California Condors in the 21st Century. Washington, D.C. and Cambridge, MA: American Ornithologists’ Union and Nuttall Ornithological Club; 2007.

[13] Walters JR, Derrickson SR, Fry DM, Haig SM, Marzluff JM, Wunderle JM Jr. Status of the California Condor (*Gymnogyps californianus*) and efforts to achieve its recovery. Auk. 2010;127:969–1001.

[14] Finkelstein ME, Doak DF, George D, Burnett J, Brandt J, Church M, et al Lead poisoning and the deceptive recovery of the critically endangered California condor. Proceedings of the National Academy of Sciences of the United States of America. 2012;109:11449–54. 10.1073/pnas.1203141109 22733770 PMC3396531

[15] D’Elia J, Haig SM. California Condors in the Pacific Northwest. Corvallis, OR: OSU Press; 2013.

[16] Meretsky VJ, Snyder NFR. Range use and movements of California condors. Condor. 1992;94:313–35.

[17] D’Elia J, Haig SM, Johnson M, Marcot B, Young R. Activity-specific ecological niche models for planning reintroductions of California condors (*Gymnogyps californianus*). Biological Conservation. 2015;184:90–9.

[18] McRae BH, Dickson BG, Keitt TH, Shah VB. Using circuit theory to model connectivity in ecology, evolution, and conservation. Ecology. 2008;89:2712–24. 10.1890/07-1861.1 18959309

[19] Cianfrani C, Maiorano L, Loy A, Kranz A, Lehmann A, Maggini R, et al There and back again? Combining habitat suitability modeling and connectivity analyses to assess a potential return of the otter to Switzerland. Animal Conservation. 2013;16:584–94.

[20] Jarchow CJ, Hossack BR, Sigafus BH, Schwalbe CR, Muths E. Modeling habitat connectivity to inform reintroductions: a case study with the Chiricahua Leopard frog. Journal of Herpetology. 2016;50:63–9.

[21] Ziółkowska E, Perzanowski K, Bleyhl B, Ostapowicz K, Kuemmerle T. Understanding unexpected reintroduction outcomes: why aren’t European bison colonizing suitable habitat in the Carpathians? Biological Conservation. 2016;195:106–17.

[22] Phillips SJ, Anderson RP, Schapire RE. Maximum entropy modeling of species geographic distributions. Ecological Modelling. 2006;190:231–59.

[23] Phillips S. A brief tutorial on Maxent. Lessons in Conservation. 2010;3:108–35.

[24] Elith J, Phillips SJ, Hastie T, Dudík M, Chee YE, Yates CJ. A statistical explanation of MaxEnt for ecologists. Diversity and Distributions. 2011;17:43–57.

[25] Cianfrani C, Le Lay G, Hirzel AH, Loy A. Do habitat suitability models reliably predict the recovery areas of threatened species? Journal of Applied Ecology. 2010;47:421–30.

[26] Merow C, Smith MJ, Edwards TC Jr., Guisan A, McMahohn SM, Normand S, et al What do we gain from simplicity versus complexity in species distribution models? Ecography. 2014;37:1267–81.

[27] U.S. Fish and Wildlife Service. Hopper Mountain National Wildlife Refuge Complex California Condor Recovery Program 2016 Annual Report. Ventura, CA: California Condor Recovery Office; 2017.

[28] Robertson PA, Aebischer NJ, Kenward RE, Hanski IK, Williams NP. Simulation and jack-knifing assessment of home-range indices based on underlying trajectories. Journal of Applied Ecology. 1998;35:928–40.

[29] Gavashelishvili A, McGrady M, Ghasabian M, Bildstein KL. Movements and habitat use by immature Cinereous vultures from the Caucasus. Bird Study. 2012;59:449–62.

[30] Phillips SJ, Dudík M, Elith J, Graham CH, Lehmann A, Leathwick J, et al Sample selection bias and presence-only distribution models: implications for background and pseudo-absence data. Ecological Applications. 2009;19:181–97. 10.1890/07-2153.1 19323182

[31] Phillips SJ, Dudík M. Modeling of species distributions with Maxent: new extensions and a comprehensive evaluation. Ecography. 2008;31:161–75.

[32] Pennycuick CJ. Power requirements for horizontal flight in the pigeon *Columba livia*. Journal of Experimental Biology. 1968;49:527–55.

[33] Poessel SA, Brandt J, Miller TA, Katzner TE. Meteorological and environmental variables affect flight behavior and decision-making of an obligate soaring bird, the California Condor *Gymnogyps californianus*. Ibis. 2017;160:36–53.

[34] Ruxton GD, Houston DC. Obligate vertebrate scavengers must be large soaring fliers. Journal of Theoretical Biology. 2004;228:431–6. 10.1016/j.jtbi.2004.02.005 15135041

[35] Duriez O, Kato A, Tromp C, Dell’Omo G, Vyssotski AL, Sarrazin F, et al How cheap is soaring flight in raptors? A preliminary investigation in free-flying vultures. PLoS ONE [Internet]. 2014;9(1):e84887 Available from: 10.1371/journal.pone.0084887 24454760 PMC3893159

[36] Koford CB. The California Condor. Research Report No. 4 ed. New York, NY: National Audubon Society; 1953.

[37] Snyder NFR, Ramey RR, Sibley FC. Nest-site biology of the California condor. Condor. 1986;88:228–41.

[38] Rivers JW, Johnson JM, Haig SM, Schwarz CJ, Glendening JW, Burnett LJ, et al Resource selection by the California condor (*Gymnogyps californianus*) relative to terrestrial-based habitats and meteorological conditions. PLoS ONE [Internet]. 2014;9(2):e88430 Available from: 10.1371/journal.pone.0088430 24523893 PMC3921182

[39] Donázar JA, Hiraldo F, Bustamante J. Factors influencing nest site selection, breeding density and breeding success in the bearded vulture (*Gypaetus barbatus*). Journal of Applied Ecology. 1993;30:504–14.

[40] Poirazidis K, Goutner V, Skartsi T, Stamou G. Modelling nesting habitat as a conservation tool for the Eurasian black vulture (*Aegypius monachus*) in Dadia Nature Reserve, northeastern Greece. Biological Conservation. 2004;118:235–48.

[41] Gavashelishvili A, McGrady MJ. Breeding site selection by bearded vulture (*Gypaetus barbatus*) and Eurasian griffon (*Gyps fulvus*) in the Caucasus. Animal Conservation. 2006;9:159–70.

[42] Mateo-Tomás P, Olea PP. Combining scales in habitat models to improve conservation planning in an endangered vulture. Acta Oecologica. 2010;35:489–98.

[43] Neveda-Rodríguez A, Vargas FH, Kohn S, Zapata-Ríos G. Andean Condor (*Vultur gryphus*) in Ecuador: geographic distribution, population size and extinction risk. PLoS ONE [Internet]. 2016;11(3):e0151827 Available from: 10.1371/journal.pone.0151827 26986004 PMC4795543

[44] Spiegel O, Getz WM, Nathan R. Factors influencing foraging search efficiency: why do scarce lappet-faced vultures outperform ubiquitous white-backed vultures. American Naturalist. 2013;181:E102–E115. 10.1086/670009 23594555

[45] Lisney TJ, Stecyk K, Kolominsky J, Graves GR, Wylie DR, Iwaniuk AN. Comparison of eye morphology and retinal topography in two species of New World vultures (Aves: Cathartidae). Anatomical Record. 2013;296:1954–70. 10.1002/ar.22815 24249399

[46] Warren DL, Seifert SN. Ecological niche modeling in Maxent: the importance of model complexity and the performance of model selection criteria. Ecological Applications. 2011;21:335–42. 10.1890/10-1171.1 21563566

[47] Trainor AM, Walters JR, Morris WF, Sexton J, Moody A. Empirical estimation of dispersal resistance surfaces: a case study with red-cockaded woodpeckers. Landscape Ecology. 2013;28:755–67.

[48] McRae BH, Shah VB. Circuitscape User’s Guide Version 3.5. Santa Barbara, CA: The University of California, Santa Barbara; 2011.

[49] Pennycuick CJ, Scholey KD. Flight behavior of Andean condors *Vultur gryphys* and Turkey Vultures *Cathartes aura* around the Paracas Peninsula, Peru. Ibis. 1984;126:253–6.

[50] Bildstein KL, Bechard MJ, Farmer C, Newcomb L. Narrow sea crossings present major obstacles to migrating Griffon Vultures *Gyps fulvus*. Ibis. 2009;151:382–91.

[51] Nourani E, Yamaguchi NM. The effects of atmospheric currents on migratory behavior of soaring birds: a review. Ornithological Science. 2017:5–15.

[52] Hirzel AH, Le Lay G, Helfer V, Randin C, Guisan A. Evaluating the ability of habitat suitability models to predict species presences. Ecological Modelling. 2006;199:142–52.

[53] Di Cola V, Broennimann O, Petitpierre B, Breiner FT, D’Amen M, Randin C, et al ecospat: an R package to support spatial analyses and modeling of species niches and distributions. Ecography. 2017;40:774–87.

[54] Koen EL, Bowman J, Sadowski C, Wapole AA. Landscape connectivity for wildlife: development and validation of multispecies linkage maps. Methods in Ecology and Evolution. 2014;5:626–33.

[55] McRae BH, Kavanagh DM. Linkage Mapper connectivity analysis software. The Nature Conservancy, Seattle WA. [Internet]. http://www.circuitscape.org/linkagemapper.

[56] Austin MP. Spatial prediction of species distribution: an interface between ecological theory and statistical modeling. Ecological Modelling. 2002;157:101–18.

[57] Randin CF, Dirnbock T, Dullinger S, Zimmermann NE, Zappa M, Guisan A. Are niche-based species distribution models transferable in space? Journal of Biogeography. 2006;33:1689–703.

[58] Elith J, Kearney M, Phillips S. The art of modelling range-shifting species. Methods in Ecology and Evolution. 2010;1:330–42.

[59] Mesgaran MB, Cousens RD, Webber BL. Here be dragons: a tool for quantifying novelty due to covariate range and correlation change when projecting species distribution models. Diversity & Distributions. 2014;20:1147–59.

[60] Moon JB, Dewitt TH, Errend MN, Bruins RJF, Kentula ME, Chamberlain SJ, et al Model application niche analysis: assessing the transferability and generalizability of ecological niche models. Ecosphere [Internet]. 2017;8(10):e01974 Available from: 10.1002/ecs2.1974. 30237908 PMC6140329

[61] Elith J, Leathwick JR. Species distribution models: ecological explanation and prediction across space and time. The Annual Review of Ecology, Evolution, and Systematics. 2009;40:677–97.

[62] Austin M. Species distribution models and ecological theory: a critical assessment and some possible new approaches. Ecological Modelling. 2007;200:1–19.

[63] Dzialak MR, Olson CV, Harju SM, Webb SL, Winstead JB. Temporal and hierarchical spatial components of animal occurrence: conserving seasonal habitat for greater sage-grouse. Ecosphere [Internet]. 2012;3(4):30 Available from: 10.1890/ES11-00315.1.

[64] Alarcón PAE, Lambertucci SA. A three-decade review of telemetry studies on vultures and condors. Movement Ecology [Internet]. 2018;6:13 Available from: 10.1186/s40462-018-0133-5. 30202527 PMC6122777

[65] Blazquez-Cabrera S, Gaston A, Beier P, Garrote G, Simon MA, Saura S. Influence of separating home range and dispersal movements on characterizing corridors and effective distances. Landscape Ecology. 2016;31:2355–66.

[66] Santos CD, Hanssen F, Munoz A, Onrubia A, Wikelski M, May R, et al Match between soaring modes of black kites and the fine-scale distribution of updrafts. Scientific Reports [Internet]. 2017;7:6421 Available from: 10.1038/s41598-017-05319-8. 28743947 PMC5526945

[67] Shamoun-Baranes JZ, Liechti F, Vansteelant WMG. Atmospheric conditions create detours and tailbacks for migrating birds. Journal of Comparative Physiology A Neuroethology Sensory Neural and Behavioral Physiology. 2017;203:509–29. 10.1007/s00359-017-1181-9 PMC5522504 28508130

[68] Leshem Y, Yom-Tov Y. Routes of migrating soaring birds. Ibis. 1998;140:41–52.

[69] Alpert P, Tannhauser DS, Leshem Y, Kravitz A, Rabinovitch-Hadar M. Migrating soaring birds align along sea-breeze fronts; first evidence from Israel. Bulletin of the American Meteorological Society. 2000;81:1599–601.

[70] Bogliani G, Viterbi R, Nicolino M. Habitat use by a reintroduced population of bearded vultures (*Gypaetus barbatus*) in the Italian Alps. Journal of Raptor Research. 2011;45:56–62.

[71] Monsarrat S, Benhamou S, Sarrazin F, Bessa-Gomes C, Bouten W, Duriez O. How predictability of feeding patches affects home range and foraging habitat selection in avian social scavengers? PLoS ONE [Internet]. 2013;8(1):e53077 Available from: 10.1371/journal.pone.0053077 23301024 PMC3536817

[72] Grigg NP, Krilow JM, Gutierrez-Ibanez C, Wylie DR, Graves GR, Iwaniuk AN. Anatomical evidence for scent guided foraging in the turkey vulture. Scientific Reports [Internet]. 2017;7:17408 Available from: 10.1038/s41598-017-17794-0 29234134 PMC5727128

[73] Lambertucci SA, Alarcón PAE, Hiraldo F, Sanchez-Zapata JA, Blanco G, Donázar JA. Apex scavenger movements call for transboundary conservation policies. Biological Conservation. 2018;170:145–150.

[74] Johnstone JA, Dawson TE. Climatic context and ecological implications of summer fog decline in the coast redwood region. Proceedings of the National Academy of Sciences of the United States of America. 2010;107:4533–8. 10.1073/pnas.0915062107 20160112 PMC2822705

[75] Ryan BC. A mathematical model for diagnosis and prediction of surface winds in mountainous terrain. Journal of Applied Meteorology. 1977;16:571–84.

[76] Ryan BC. WNDCOM: estimating surface winds in mountainous terrain. General Technical Report PSW-73. Berkeley, CA: Pacific Southwest Forest and Range Experiment Station; 1983.

[77] Bakker VJ, Smith DR, Copeland H, Brandt J, Wolstenholme R, Burnett J, et al Effects of lead exposure, flock behavior, and management actions on the survival of California condors (Gymnogyps californianus). EcoHealth [Internet]. 2016; 10.1007/s10393-015-1096-2 26769426

[78] Galef BG, Laland KN. Social learning in animals: empirical studies and theoretical models. BioScience. 2005;55:489–99.

[79] Morandini V, Ferrer M. Natal philopatry: local experience or social attraction? An experiment with Spanish imperial eagles. Animal Behaviour. 2017;130:153–7.

[80] Ventana Wildlife Society. Ventana Wildlife Society’s California Condor Program 2015 Annual Report. Salinas, CA: Ventana Wildlife Society; 2016.

[81] Ventana Wildlife Society. Ventana Wildlife Society’s California Condor Program 2016 Annual Report. Salinas, CA: Ventana Wildlife Society; 2017.

[82] Álvarez M, Gálvez M, Millet A, Marco X, Álvarez E, Rafa M, et al “Vulturnet” connectivity of the European populations of Cinereous Vulture: A programme to reintroduce the species into Catalonia In: Zuberogoitia I, Martínez JE, editors. Ecology and Conservation of European Forest-Dwelling Raptors. Bilbao, Spain: Departamento de Agricultura de la Diputación Foral de Bizkaia; 2011 p. 356–61.

[83] Stoynov E, Peshev H, Grozdanov A, Vangelova N. Five years overview of the reintroduction of Griffon Vulture *Gyps fulvus* in Kresna Gorge, Bulgaria. Vulture News. 2015;69:33–9.

[84] Schumaker NH, Brookes A. HexSim: a modeling environment for ecology and conservation. Landscape Ecology. 2018;33:197–211. 10.1007/s10980-017-0605-9 29545713 PMC5846496

[85] Nathan R, Getz WM, Revilla E, Holyoak M, Kadmon R, Saltz D, et al A movement ecology paradigm for unifying organismal movement research. Proceedings of the National Academy of Sciences of the United States of America. 2008;105:19052–9. 10.1073/pnas.0800375105 19060196 PMC2614714

[86] Miller ML, Ringelman KM, Eadie JM, Schank JC. Time to fly: A comparison of marginal value theorem approximations in an agent-based model of foraging waterfowl. Ecological Modelling. 2017;351:77–86.

[87] Mihoub J, Le Gouar P, Sarrazin F. Breeding habitat selection behaviors in heterogeneous environments: implications for modeling reintroduction. Oikos. 2009;118:663–74.

[88] Rytteri S, Kuussaari M, Saastamoinen M, Ovaskainen O. Can we predict the expansion rate of a translocated butterfly population based on a priori estimated movement rates? Biological Conservation. 2017;215:189–95.

[89] Gilroy JJ, Lockwood JL, Both C. Simple settlement decisions explain common dispersal patterns in territorial species. Journal of Animal Ecology. 2016;85:1182–90. 10.1111/1365-2656.12545 27155215

[90] Ovaskainen O, Rekola H, Meyke E, Arjas E. Bayesian methods for analyzing movements in heterogeneous landscapes from mark-recapture data. Ecology. 2008;89:542–54. 10.1890/07-0443.1 18409443

[91] Plank MJ, Codling EA. Sampling rate and misidentification of Lévy and non-Lévy movement paths. Ecology. 2009;90:3546–53. 10.1890/09-0079.1 20120821

[92] Lookingbill TR, Gardner RH, Ferrari JR, Keller CE. Combining a dispersal model with network theory to assess habitat connectivity. Ecological Applications. 2010;20:427–41. 10.1890/09-0073.1 20405797

[93] Richardson KM, Doerr V, Ebrahimi M, Lovegrove TG, Parker KA. Chapter 6: Considering dispersal in reintroduction and restoration planning In: Armstrong DP, Hayward MW, Moro D, Seddon PJ, editors. Advances in Reintroduction Biology of Australian and New Zealand Fauna. Melbourne, Australia: CSIRO Publishing; 2015 p. 59–72.

[94] U.S. Fish and Wildlife Service. Endangered and threatened wildlife and plants; experimental populations. Federal Register. 1984;49:33885–94.

[95] Sainsbury AW, Vaughan-Higgins RJ. Analyzing disease risks associated with translocations. Conservation Biology. 2012;26:442–52. 10.1111/j.1523-1739.2012.01839.x 22533691

[96] Weeks AR, Sgro CM, Young AG, Frankham R, Mitchell NJ, Miller KA, et al Assessing the benefits and risks of translocations in changing environments: a genetic perspective. Evolutionary Applications. 2011;4:709–25. 10.1111/j.1752-4571.2011.00192.x 22287981 PMC3265713

[97] D’Elia J, Haig SM, Mullins T, Miller MP. Ancient DNA reveals substantial genetic diversity in the California Condor (*Gymnogyps californianus*) prior to a population bottleneck. Condor. 2016;118:703–14.

[98] Southwest Condor Working Group. California Condor Recovery Program in the Southwest; Fourth Review (2012–2016). Phoenix, AZ: U.S. Fish and Wildlife Service Arizona Ecological Services Office; 2017.

[99] Zamboni T, Di Martino S, Jiménez-Pérez I. A review of multispecies reintroduction to restore a large ecosystem: The Iberá Rewilding Program (Argentina). Perspectives in Ecology and Conservation. 2017;15:248–56.

