# Supplementary material for: Applying circuit theory and landscape linkage maps to reintroduction planning for California Condors
Source: PLoS One. 2019 Dec 31;14(12):e0226491. doi: 10.1371/journal.pone.0226491 (PMC6938332; doi:10.1371/journal.pone.0226491)
Supplement: S1 Table — (DOCX) [file pone.0226491.s001.docx]

**S1 Table.** California condors outfitted with Global System for Mobile Communications transmitters from July 2013 to May 2017, California, USA.

| **Studbook #** | **Movebank Dataset**^1^ | **Hatch Year** | **Sex** | **Earliest date**^2^ | **Latest date**^2^ | **Number of Movement Locations**^3^ | **Days of Data**^3^ |
| --- | --- | --- | --- | --- | --- | --- | --- |
| 20 | FWS | 1980 | Male | 2016-01-04 | 2017-05-31 | 4,548 | 376 |
| 21 | FWS | 1980 | Male | 2014-05-07 | 2016-06-24 | 10,956 | 667 |
| 63 | FWS | 1991 | Male | 2014-06-18 | 2014-09-28 | 323 | 36 |
| 76 | FWS | 1992 | Female | 2017-02-12 | 2017-05-31 | 275 | 19 |
| 77 | FWS | 1992 | Female | 2017-03-20 | 2017-05-31 | 6,148 | 41 |
| 79 | FWS | 1992 | Female | 2014-06-25 | 2017-05-31 | 6,991 | 566 |
| 98 | FWS | 1994 | Male | 2014-02-12 | 2016-07-31 | 144,658 | 840 |
| 107 | FWS | 1994 | Male | 2014-06-16 | 2017-05-31 | 60,519 | 665 |
| 147 | FWS | 1996 | Female | 2015-01-15 | 2016-04-06 | 4,530 | 341 |
| 156 | FWS | 1997 | Female | 2016-05-21 | 2017-05-31 | 42,616 | 230 |
| 161 | FWS | 1997 | Female | 2014-06-24 | 2017-05-31 | 45,359 | 751 |
| 180 | FWS | 1998 | Female | 2015-06-17 | 2016-01-08 | 1,091 | 148 |
| 192 | FWS | 1998 | Female | 2014-07-18 | 2015-09-12 | 5,879 | 377 |
| 237 | FWS | 2001 | Male | 2014-12-25 | 2016-01-21 | 3,955 | 263 |
| 247 | FWS | 2001 | Male | 2016-05-21 | 2017-05-28 | 2,252 | 197 |
| 255 | FWS | 2001 | Female | 2014-07-12 | 2015-12-21 | 6,262 | 414 |
| 262 | FWS | 2001 | Male | 2013-12-04 | 2016-07-19 | 117,930 | 837 |
| 289 | FWS | 2002 | Female | 2014-11-06 | 2017-05-31 | 12,578 | 803 |
| 326 | FWS | 2004 | Male | 2014-02-04 | 2016-07-20 | 120,244 | 804 |
| 360 | FWS | 2005 | Male | 2015-10-20 | 2017-04-08 | 4,539 | 370 |
| 365 | FWS | 2005 | Male | 2014-06-11 | 2016-05-26 | 90,371 | 611 |
| 370 | FWS | 2014 | Female | 2013-11-22 | 2014-09-26 | 34,452 | 270 |
| 374 | FWS | 2005 | Male | 2014-07-19 | 2017-01-08 | 16,338 | 656 |
| 449 | FWS | 2007 | Female | 2014-06-26 | 2017-05-31 | 8,598 | 840 |
| 480 | FWS | 2008 | Male | 2014-11-06 | 2017-05-31 | 75,171 | 540 |
| 482 | FWS | 2008 | Male | 2013-12-04 | 2014-11-29 | 34,193 | 304 |
| 483 | FWS | 2008 | Female | 2016-06-08 | 2016-07-31 | 12,920 | 54 |
| 487 | FWS | 2008 | Female | 2014-11-06 | 2016-10-05 | 4,123 | 486 |
| 493 | FWS | 2008 | Female | 2014-01-25 | 2016-07-29 | 125,149 | 856 |
| 507 | FWS | 2009 | Female | 2016-08-09 | 2017-03-31 | 40,995 | 178 |
| 509 | FWS | 2009 | Male | 2014-12-13 | 2017-05-31 | 8,547 | 637 |
| 513 | FWS | 2009 | Female | 2014-01-08 | 2017-05-31 | 100,826 | 1,042 |
| 518 | FWS | 2009 | Female | 2013-12-18 | 2016-11-24 | 5,758 | 680 |
| 526 | FWS | 2009 | Female | 2014-05-20 | 2017-05-27 | 8,077 | 821 |
| 560 | FWS | 2010 | Female | 2014-06-25 | 2016-01-22 | 5,990 | 512 |
| 570 | FWS | 2010 | Male | 2017-03-08 | 2017-05-31 | 9,773 | 77 |
| 584 | FWS | 2010 | Female | 2016-05-22 | 2016-07-19 | 6,322 | 53 |
| 585 | FWS | 2010 | Male | 2015-06-10 | 2017-05-31 | 9,706 | 638 |
| 599 | FWS | 2011 | Female | 2014-02-21 | 2016-11-03 | 5,397 | 519 |
| 625 | FWS | 2011 | Male | 2014-06-11 | 2017-05-31 | 99,673 | 741 |
| 636 | FWS | 2012 | Male | 2014-09-17 | 2017-05-31 | 5,981 | 754 |
| 648 | FWS | 2012 | Female | 2014-06-24 | 2016-12-31 | 9,463 | 796 |
| 683 | FWS | 2013 | Male | 2015-06-28 | 2016-06-23 | 3,301 | 295 |
| 717 | FWS | 2013 | Female | 2014-07-20 | 2015-09-20 | 4,294 | 366 |
| 733 | FWS | 2014 | Male | 2015-07-28 | 2017-05-31 | 4,284 | 466 |
| 791 | FWS | 2015 | Female | 2016-06-03 | 2017-05-31 | 38,010 | 314 |
| 231 | PINN | 2000 | Female | 2015-11-12 | 2016-02-14 | 405 | 50 |
| 411 | PINN | 2006 | Male | 2014-11-23 | 2014-12-03 | 94 | 6 |
| 431 | PINN | 2007 | Male | 2014-08-31 | 2015-02-06 | 17,178 | 150 |
| 460 | PINN | 2008 | Female | 2014-09-01 | 2014-11-15 | 6,552 | 50 |
| 525 | PINN | 2009 | Female | 2015-06-18 | 2017-05-31 | 6,958 | 457 |
| 534 | PINN | 2009 | Female | 2015-10-26 | 2016-10-13 | 2,391 | 288 |
| 543 | PINN | 2009 | Female | 2015-10-21 | 2016-10-31 | 2,326 | 250 |
| 547 | PINN | 2009 | Female | 2015-06-05 | 2017-05-29 | 53,592 | 337 |
| 550 | PINN | 2010 | Female | 2015-11-22 | 2016-06-05 | 88 | 16 |
| 564 | PINN | 2010 | Male | 2014-10-17 | 2017-05-31 | 101,794 | 731 |
| 589 | PINN | 2010 | Male | 2015-03-04 | 2015-06-30 | 492 | 76 |
| 597 | PINN | 2011 | Female | 2015-10-07 | 2017-05-31 | 3,630 | 334 |
| 614 | PINN | 2011 | Female | 2016-03-27 | 2017-04-26 | 20,247 | 228 |
| 615 | PINN | 2011 | Male | 2015-10-30 | 2015-11-11 | 148 | 11 |
| 687 | PINN | 2013 | Female | 2015-03-10 | 2017-05-07 | 4,755 | 475 |
| 477 | VEN | 2008 | Male | 2013-07-04 | 2016-10-04 | 65,799 | 546 |
| 567 | VEN | 2010 | Male | 2013-07-27 | 2016-10-11 | 81,261 | 670 |
| 678 | VEN | 2013 | Female | 2016-11-18 | 2017-05-31 | 6,842 | 72 |
| 696 | VEN | 2013 | Male | 2015-11-30 | 2017-04-15 | 32,878 | 323 |
| 697 | VEN | 2013 | Male | 2015-08-04 | 2017-05-31 | 24,660 | 207 |
| 703 | VEN | 2013 | Male | 2015-11-29 | 2017-05-31 | 34,798 | 300 |
| 706 | VEN | 2013 | Male | 2015-11-03 | 2017-05-31 | 33,018 | 266 |
| 711 | VEN | 2013 | Male | 2016-02-03 | 2016-10-22 | 28,181 | 201 |
| 716 | VEN | 2013 | Male | 2015-12-11 | 2016-10-22 | 26,228 | 202 |
| 718 | VEN | 2013 | Male | 2015-08-04 | 2017-05-31 | 38,840 | 335 |
| 726 | VEN | 2014 | Female | 2017-03-01 | 2017-05-29 | 6,694 | 64 |
| 747 | VEN | 2014 | Female | 2016-12-04 | 2017-05-29 | 5,352 | 54 |
| 758 | VEN | 2014 | Male | 2017-01-31 | 2017-05-31 | 8,989 | 77 |
| 760 | VEN | 2014 | Female | 2017-02-12 | 2017-05-31 | 8,540 | 63 |

^1^ FWS = Fish and Wildlife Service, PINN = Pinnacles National Park, VEN = Ventana Wildlife Society

^2^ Earliest and latest dates refer to the earliest and latest date that GSM occurrence data were available for that individual in our dataset that was filtered to remove stationary points, points with recorded speeds >30 m/s, points that were not “proofed”, points that were offshore, and points within 5 km of release sites and the Hopper Mountain NWR flight pen.

^3^ The number of movement locations and number of days of data refer to the filtered dataset (see footnote 2).
